# Children’s mental representations with respect to caregivers and post-traumatic symptomatology in Somatic Symptom Disorders and Disruptive Behavior Disorders

**DOI:** 10.3389/fpsyg.2015.01125

**Published:** 2015-08-03

**Authors:** Fabiola Bizzi, Donatella Cavanna, Rosetta Castellano, Cecilia S. Pace

**Affiliations:** Department of Educational Science, University of GenoaGenoa, Italy

**Keywords:** mental representations, post-traumatic symptomatology, parent–child relationship, disorganized attachment, Somatic Symptom Disorder, Disruptive Behavior Disorder

## Abstract

**Introduction:** In line with literature, the quality of adult–infant interactions and mental representations of the caregivers play an essential role in influencing the children’s well-being. Many studies focused the attention on the role of attachment for a better evaluation of child psychopathological outcomes. The flexibility of the child’s attachment model gives the opportunity to parents to be helped in modifying their own caregiving quality, encouraging the reflection on the children’s state of mind with respect to attachment. The aims of this study were to evaluate: (1) the attachment models in young patients diagnosed with Disruptive Behavior Disorders (DBDs) and Somatic Symptoms Disorders (SSDs); (2) the levels of post-traumatic symptomatology; (3) the association between the attachment models and post-traumatic symptomatology.

**Methods:** Forty Italian patients, aged from 8 to 15, recruited at Gaslini Paediatric Hospital of Genoa, previously diagnosed with SSD (*N* = 20) and DBD (*N* = 20) were assessed using the Child Attachment Interview (CAI), the Separation Anxiety Test (SAT), the Trauma Symptom Checklist for Children (TSCC-A). Socio-demographic data were collected.

**Results:** In both the clinical samples, the findings on the distribution of attachment models showed a significant presence of insecure attachment with respect to both parents in more than a half of the patients and high levels of disorganized attachment. No significant differences between DBD and SSD samples were found on post-traumatic symptomatology (Post-Traumatic Stress and Dissociation). Significant differences were found on Depression, Anxiety, and Fantasy subscales.

**Discussion:** This study can provide a detection of dysfunctional aspects in clinical populations. The findings suggest that the quality of the attachment to parents may be a fundamental element to better assess SSD and DBD in children and adolescents. Clinical implications of this study aimed at improving parental caregiving are highlighted.

## Introduction

One of the widely documented findings on child psychopathology is the consistent relation between the quality of parental caregiving and child psychopathological outcomes in terms of externalizing as well as internalizing problems (Campbell et al., 2010; Manongdo and García, 2011; Wang et al., 2013). Studies have found empirical support to the most influential theories of etiological contributors to child psychopathology, considering three main dimensions (Lecompte and Moss, 2014; Yap and Jorm, 2015): (1) parental aspects, e.g., parenting style and parental interaction; (2) child characteristics, e.g., temperament, neurobiological aspects; (3) attachment relationship quality.

The extensive research on attachment provides a scientific foundation for positing relational as well as biological contributors to many forms of child psychopathological outcomes as well as their association with parent–child relationship and parental qualities of care, such as availability, or neglect, rejection, etc. In line with the growing literature on the neurobiological correlates of attachment, the links among parental maladaptive caregiving, children’s attachment disorganization, and psychopathological outcomes, are current objects of investigation.

Early attachment experiences have a long-term impact on the child’s mental health because the emotional and behavioral regulatory patterns developed within the parent–child relationship influence the way children express their emotions and behaviors (Groh et al., 2012). Caregivers guide their children in exploring emotions and thoughts, thereby assisting them in the organization of their emotional experience, and in the development of social abilities (Thompson, 2008). Moreover, parent–child interactions shape children’s ‘Internal Working Models’ (IWMs), defined as script-like representations of secure base experience (Bowlby, 1982). Attachment patterns are adaptive responses to a caregiving environment and are designed to promote the child’s safety.

It is attested that positive aspects of parenting, such as warmth, positive involvement, and secure child–parent attachment are linked to psychological well-being and positive adaptation of the child (Boeldt et al., 2012). Studies have shown that children who have experienced supportive and sensitive care and positive interactions with their parents are more likely to develop a secure attachment (Cyr et al., 2010; Dubois-Comtois et al., 2011, 2013). They are also more socially adapted and tend to use more effective emotion regulation strategies than their insecure peers (Easterbrooks et al., 2012). Other studies have found that the parents’ ability to manage the child’s behavior while responding in an emotionally attuned way is associated with lower levels of child negative behavioral and more positive socio-emotional outcomes (Mikulincer and Shaver, 2012).

Alternatively, children experiencing unavailable care and involved in disrupted parent–child interactions are more likely to develop insecure attachment and emotional dysregulation (Azak et al., 2012). It is attested that experiences of neglectful and frightening care in childhood can be considered chronic interpersonal traumas. These experiences leave the child with little possibility of attaining affective security (Carpenter and Chung, 2011) because, in these situations, the caregiver is often both the source of alarm and the one who should be providing comfort. These conditions compromise the child’s ability to predict parental behavior and to develop his/her sense of efficacy. Among these insecure attachment patterns, the disorganized one is considered to be the most at-risk for developmental psychopathology (Solomon and George, 2011). Children showing a disorganized attachment are unable to successfully use their caregivers to regulate emotion and behavior, having experienced traumatic states, such as frightening and/or frightened behavior in their distressed caregivers (Lyons-Ruth and Jacobvitz, 2008; Scott et al., 2011).

The relationship between attachment disorganization and dissociative symptomatology is consistent throughout the lifespan (West et al., 2001; Haltigan and Roisman, 2015). Researchers have reported three main frequent elements in children with disorganized attachment (Hesse and Main, 2000; Liotti, 2011): (1) the collapse of the controlling strategies; (2) the reactivation of the disorganized IWM in the form of bizarre and contradictory behaviors; (3) the possibility to be caught in dissociative experiences. Moreover, literature shows that multiple exposures to trauma and ruptures of attachment relationships are associated with *post-traumatic symptoms*, including difficulties in the regulation of affect and behavior, anger, anxiety, depression, post-traumatic stress, dissociation and somatization (Putnam, 1997; Dutra et al., 2009; Kugler et al., 2012; Zaccagnino et al., 2015).

While literature on the influence of quality of parental care on child development and psychopathology in infancy and childhood is widespread, the investigation of attachment relationships in middle childhood (usually aged 8–12 years-old) and early adolescence (13–15 years-old) shows inconsistent data. Middle childhood is a particularly challenging period in which parental caregiving is subject to numerous transformations. In this period, parents have to face challenges arising from both maturational changes, and from new social demands (Collins et al., 2005). According to Collins et al. (2005), “these changes inevitably alter the amount, kind, content, and significance of interaction between parents and children” (p. 73). The most important changes are due to: (1) the growth in knowledge and in cognitive competences in children, (2) the expansion of social networks and the new value of relationships with peers (Kerns and Richardson, 2005). However, it is attested that, in middle childhood, the perception of parents as sources of both emotional support and instrumental help remains typically stable during this age.

Studies on attachment during middle childhood and early adolescence confirm that secure attachment is associated with greater resilience and lower levels of internalizing and externalizing problems (Fearon and Belsky, 2011; Groh et al., 2012). On the contrary, attachment disorganization and controlling strategies are linked to higher levels of anxiety and maladaptive socio-emotional functioning (Goldwyn et al., 2000; Moss and St-Laurent, 2001; Moss et al., 2006). As Moss et al. (2006) stated: “there is still considerable ambiguity concerning associations between attachment and behavior problem risk, particularly with respect to the role of different insecure classifications in predicting level and type of problem behavior. This is particularly true of middle childhood, a period during which fewer studies have been conducted and in which none have used a comprehensive self-report measure of behavior problems” (p. 428).

Several aspects could explain the paucity of studies on middle childhood (Kerns, 2008). First, attachment in middle childhood can be investigated using narratives [e.g., the Child Attachment Interview (CAI)], projective tests [such as the Separation Anxiety Test (SAT)] and questionnaires (such as the Security Scale). These measures capture different attachment dimensions (e.g., representations, perceptions, behaviors). Second, another source of inconsistent data in literature is the use of perceptions of child behavior from their significant adults (e.g., mother, teacher). Studies have shown that external observers may underestimate the internalizing symptoms because of the children’s reluctance to share these problems; or, on the contrary, they may overestimate externalizing symptoms (Youngstrom et al., 2000). Third, another critical aspect is that those measures considered to be appropriate during the latter half of middle childhood may not be equally sensitive and valid at younger ages, and vice versa. Finally, several attachment measures fail to catch attachment disorganization, the attachment pattern which is more capable to predict subsequent psychopathological outcomes. For these reasons, a multi-level assessment becomes particularly important for the detection of psychopathological outcomes in middle childhood.

In summing up, the attachment framework has the potential to give further understanding of the relationship between interpersonal trauma and psychopathology in the middle childhood (Fowler et al., 2013). The purpose of this study is to investigate attachment representations and post-traumatic symptoms in two groups of children: children with an *internalizing* problem, Somatic Symptom Disorder (SSD); and children with an *externalizing* problem, Disruptive Behavior Disorder (DBD). SSD is characterized by multiple and variable physical symptoms without demonstrable pathophysiological processes, accompanied by thoughts, feelings, and unusual behaviors in response to symptoms. Neurologic symptoms that are not identified by a clear organic cause, as well as psychogenic headaches and generalized pain are included in this category (American Psychiatric Association [APA], 2013). Literature has shown that this psychopathological disorder is associated with insecure attachment in childhood, especially with Insecure-Resistant attachment style (Waller et al., 2004; Kozlowska and Williams, 2009). Moreover, it has been sustained that disorganized attachment is moderately associated with internalizing symptoms (Borelli et al., 2010; Brumariu and Kerns, 2010; Madigan et al., 2013). DBD includes Oppositional-Defiant Disorder and Conduct Disorder, and involves several problematic behaviors and antisocial activities (American Psychiatric Association [APA], 2013). It is associated with Avoidant attachment style (DeKlyen and Greenberg, 2008; Fearon et al., 2010; Fearon and Belsky, 2011). Other research findings have indicated the role of attachment disorganization in predicting externalizing problems in infancy and middle childhood (Green et al., 2007; Bureau et al., 2009; Borelli et al., 2010).

The objectives of the present study are:

(1) To evaluate the attachment representations with respect to parents in two middle childhood and early adolescence risks samples. Our hypotheses are to find*: (a) an over-representation of Insecure and Disorganized attachment in the overall sample; (b) an over-representation of Preoccupied attachment in SSD subjects than in DBD subjects; (c) an over-representation of Dismissing attachment in DBD subjects than in SSD subjects.*
(2) To evaluate post-traumatic symptomatology in these two groups of clinical samples. Our hypotheses are that *compared to DBD sample, SSD sample would show marked levels of post-traumatic symptomatology, in areas like Anxiety, Depression, Anger, Post-Traumatic Stress, and Dissociation*.
(3) To investigate the relation between attachment models and post-traumatic symptomatology. Our hypothesis is that *subjects with Disorganized attachment would report higher levels of post-traumatic symptomatology.*

## Materials and Methods

### Participants

The participants were 40 children and adolescents, previously diagnosed with SSDs (*n* = 20) and DBDs (*n* = 20), according to DSM 5 criteria (American Psychiatric Association [APA], 2013) by three child mental health specialists. The present study adopted the following *exclusion* criteria: (a) diagnosis of any psychotic disorder and/or (b) mental retardation, (c) drug treatment or psychotherapeutic treatment. Inclusion criteria were the age between 8 and 15, and fluency in the Italian language.

All the participants were Italian, born and living in the North–West of Italy. SSD participants were 50% female and their average age was 11.99 (SD = 2.25). DBD participants were 20% female and their average age was 11.35 (SD = 1.90). 80% of SSD and 75% of DBD subjects were living with both parents. 25% of SSD mothers and 15% of SSD fathers had a college degree. Similarly, 20% of DBD mothers and 10% of DBD fathers had a college degree. The socio-economic status (SES) of the samples was similar: in both groups 65% of the parents had a SES between 15000 and 36000 euros.

### Measures

*Child Attachment Interview* (Target et al., 2003) is a semi-structured interview designed to assess the youth’s mental representations with respect to their parental attachment figures. The youth is asked to describe relationship qualities with mother and father (e.g., “Can you tell me three words to describe your relationship with your mum, what it’s like to be with her?”), what happens when the parent gets angry with the youth, when the youth is ill, when hurt and when upset, and to provide specific examples of each scenario. It is conceptually based on the Adult Attachment Interview (AAI; George et al., 1985). The interview is videotaped and transcribed verbatim.

The CAI coding and classification system comprises of different subscales, all designed to assess the child’s overall current state of mind with respect to attachment, as reflected in both narrative and non-verbal behavior. The subscales include emotional openness, balance of positive and negative reference to attachment figures, Use of Examples, Involving Anger, Idealization, Dismissal, Resolution of Conflicts, and Overall Coherence. A score between 1 and 9 is assigned for each of the scales, based on a careful analysis of the narrative. According to the scoring on these subscales, the child’s attachment classification with respect to each caregiver is established, on “two way*”* classification (Secure-Insecure), “three way*”* classification (Secure, Dismissing, Preoccupied) and/or “four way*”* classification (Secure, Dismissing, Preoccupied, Disorganized). Each youth is assigned to one attachment classification for each parent.

This interview has previously been used with clinical and non-clinical populations (Target et al., 2003). High test–retest reliability of both scale scores and attachment classifications was demonstrated 3 months (α’s 0.74–1.00) and 1 year later (α’s 0.72–0.79). Internal consistency of the scale scores (α’s ranged from 0.84 to 0.92 for two-way) inter-rater reliability (0.92 for two-way) and validity of the measure have been determined (Target et al., 2003; Shmueli-Goetz et al., 2008). CAI classifications correlated with the child’s attachment security as measured in the SAT, maternal AAI classification and measures of social functioning (Shmueli-Goetz et al., 2008). CAI classifications are not related to age, sex, SES, ethnicity, verbal IQ, expressive language ability or whether the child lives with one parent or two (Target et al., 2003).

In this study, the interviews were separately coded by two independent coders who were trained by Shmueli-Goetz et al. (2008), and had obtained reliability certification. Coefficient kappa was calculated as an estimate of agreement. For two-way classifications with respect to the mother (Secure- Insecure) the coders’ agreement was 91.6% (κ = 0.79, *p* < 0.00). For two way classifications with respect to the father, the coders’ agreement was 92,3% (κ = 0.81, *p* < 0.00).

*Separation Anxiety Test* (Klagsbrun and Bowlby, 1976; Attili, 2001) is a semi-projective test for children and adolescents designed to assess children’s responses to scenes depicting separations from their parents. It consists of six pictures, which were labeled as “mild” or “severe” separations on the basis of the existing scoring system. The examiner describes what happens before each separation, and then follows up with questions about what the pictured child feels, why the child feels that way, and what the child will do. The pictures are gender-based. The child’s responses to the SAT are audiotaped and transcribed verbatim.

In this study, the procedure used to code SAT is the Attili (2001) coding system. A scoring ranging from -2 to +2 is attributed to each of the following eight subscales: Attachment, Loss of Self-esteem, Hostility, Trust himself, Avoidance, Anxiety, Anguish, and Confusion. On the basis of the scorings on these subscales, one of the following attachment classifications is established: Secure, Ambivalent-Anxious, Anxious-Avoidant, Disorganized, and Confused attachment.

The SAT is widely used and has good psychometric properties, including convergent validity and discriminant validity, internal consistency, and predictive validity (Attili, 2001). To test its concurrent validity, the Attili’s modified Italian version of SAT was compared with the test by Klagsbrun and Bowlby (1976). Within a sample of 83 subjects (4.4–9.3 years-old) Spearman test showed a correlation coefficient *r* = 0.77 (*p* < 0.00). Predictive validity calculated on 44 children and their mothers revealed an agreement with maternal attachment representations assessed by the AAI of 87.8% (κ = 0.67, *p* < 0.00) on the security-insecurity dimensions; and of 80.8% (κ = 0.57, *p* < 0.01) on the AAI “Unresolved state of mind” and the SAT “Disorganization” (Zaccagnino et al., 2005). Test–retest reliability was calculated on 18 subjects who were tested twice with an interval of 2 months. The Spearman coefficient for the overall scores of the four attachment categories was *r* = 0.75 (*p* < 0.00; Attili, 2001).

In this study, two independent coders scored the test. The coders agreement was of 82% (κ = 0.67, *p* < 0.00).

*Trauma Symptom Checklist for Children* (TSCC-A; Briere, 1996) is a 44-item child-report evaluating the post-traumatic symptomatology of children between 8 and 16 years-old. The detection of a cluster of psychological consequences that might have been triggered by traumatic events, such as physical and sexual abuse, major loss, peer-to-peer bullying, and experiencing the ravages of natural disaster is studied. The child is asked to score responses on a four-point Likert Scale ranging from 0 to 3. Two validity scales (Under-response and Hyper-response) and five clinical scales (Anxiety, Depression, Anger, Post-Traumatic Stress, and Dissociation) are obtained.

The Anxiety scale includes items that measure the level of the child’s generalized anxiety, hyperactivity, worries, and fear. High scores would reveal either anxiety or hyperactivity problems related to post-traumatic stress disorder. The Depression scale consists of items pertaining feelings of sadness, unhappiness, loneliness, etc., while the Anger scale is made up of items detecting feelings, thoughts and behavior expressing anger; high scores would show the presence of aggressive and hostile behaviors. The items of the Post-Traumatic-Stress scale are referred to specific post-traumatic symptoms such as intrusive thoughts, sensations and memories of early sorrowful events that can cause either anxious distraction or irritability in the children. The Dissociation scale has two subscales: Fantasy and Overt Dissociation. This scale captures the possible dissociative symptomatology; high scores would display diminished sensitiveness toward the environment, emotional detachment and the tendency to remove any affective aspect at cognitive level.

The questionnaire has been translated and validated in Italian (Di Blasio et al., 2011). It has good psychometric properties including convergent and discriminant validity (Lanktree et al., 2008), internal consistency and predictive validity (Sadowski and Friedrich, 2000). In particular, the instrument demonstrates a good level of validity (range from 0.55 to 0.88) and each scale shows adequate internal reliability (average α = 0.85).

### Procedure

Recruitment of the samples was carried out at Gaslini Hospital of Genoa (Italy). The study was previously approved by the Gaslini (IRCSS) Ethics Committee and data was collected for a whole year (2014). All participants and their families were informed about the aims and the procedures of the study. They submitted their written informed consent and were advised about their option of withdrawal at any time. The child’s assessment was conducted in a private room at the hospital and the duration of a single meeting was about 1 h and 15 min; in the same meeting in another room, parents were answering questions to collect data on socio-demographic variables. At the end of the assessment, we offered a report with a synthesis of the outcomes of each measure to the participants who completed the whole procedure. None of the children was in any kind of psychotherapeutic treatment or drug treatment at the time of the study. No case of dropout was registered.

Participation was voluntary and data was kept confidential by replacing the participants’ names with an alphanumeric code. All procedures and materials complied with the official directions established by the American Psychological Association. This study was part of a larger research project that investigates family and individual characteristics in child psychopathology.

### Statistical Analysis

The data was analyzed using the Statistical Package for the Social Science (SPSS, Version 21.0; IBM Corp., Armonk, NY, USA). Demographic variables were described using descriptive statistics (frequencies and percentages for the categorical variables, and mean and standard deviation for the continuous variables). Frequency analysis was used to test nominal and categorical variable distribution; the chi-square test and the Fisher exact test were used to test nominal and categorical variables; the independent sample test was used to compare the means in two independent samples. Results were considered statistically significant when ‘*p’* was ≤ 0.05.

## Results

Preliminary analyses were addressed to determine the possible presence of significant differences between the two groups on the socio-demographic variables. No significant differences were found between SSD and BDB samples, with respect to the socio-demographic variables of ‘age,’ ‘sex,’ ‘type of family’ (participants living with both parents, parents divorced, a parent deceased, etc.), ‘SES,’ ‘parent’s age,’ and ‘parent’s educational level’ (see **Table 1**). Only the ‘family composition’ (number of components in the family) showed marginally statistically differences between the two groups. Specifically, BDB sample consisted of more only child (50%) than SSD sample (15%; Fisher Exact Test, *p* = 0.06).

**Table 1:** Socio-demographic characteristics of the two sample groups (DBD and SSD).

Characteristic	DBD sample (*n* = 20)	SSD sample (*n* = 20)	Independent samples *t*-test/Fisher’s exact test
Mean age in years (SD)	11.35 (1.90)	11.99 (2.25)	*t*(38) = -0.97, *p* = 0.34, *ns*
% Boys	75	50	*F*(1,40) = 0.19, *ns*
%Traditional family	75	80	*F*(2,40) = 0.29, *ns*
% Only children	50	15	*F*(4,40) = 0.06, *ns*
% Middle class (SES = 15000–36000)	65	65	*F*(3,40) = 1.00, *ns*
Mother’s mean age in years (SD)	47 (14.12)	43 (9.90)	*t*(36) = -1.42, *p* = 0.16, *ns*
% Mother’s education (degree)	25	20	*F*(5,40) = 0.48, *ns*
Father’s mean age in years (SD)	49 (8.48)	46 (15.56)	*t*(34) = -0.74, *p* = 0.46, *ns*
% Father’s education (degree)	15	10	*F*(4,40) = 0.75, *ns*

### Attachment Representations with Respect to Parents in SSD and DBD Samples

In the evaluation of the attachment representations in these two middle childhood and early adolescence risk samples, we aimed at verifying: (1) the presence of an over-representation of Insecure and Disorganized attachment in the overall sample; (2) the presence of an over-representation of Preoccupied attachment in SSD subjects than in DBD subjects; (3) the presence of an over-representation of Dismissing attachment in DBD subjects than in SSD subjects.

In the overall sample of 40 clinical subjects, the majority of children were classified as Insecure with respect to both mother and father (75 and 71.8%, respectively). On the three-way classification (Secure, Dismissing, Preoccupied), a predominance of the Dismissing classification was found (50% for mother and 51.3% for father). The frequency of Preoccupied attachment was of 25% with respect to mother and 20.5% with respect to father. On the four-way classification (Secure, Dismissing, Preoccupied, Disorganized), children classified as Disorganized were 45% with respect to mother and 43.6% with respect to father (see **Table 2**). This high presence of an over-representation of Insecure and Disorganized attachment in the overall sample confirmed our first hypothesis.

**Table 2:** Distribution of attachment classification (four-way) in both samples (DBD and SSD).

	Attachment classification with respect to mother				Attachment classification with respect to father			
Samples	Secure*n* (%)	Dismissing *n* (%)	Preoccupied *n* (%)	Disorganized *n* (%)	Secure *n* (%)	Dismissing *n* (%)	Preoccupied *n* (%)	Disorganized *n* (%)
DBD (*n* = 20)	4 (20)	5 (25)	2 (10)	9 (45)	5 (25)	5 (25)	1 (5)	9 (45)
SSD (*n* = 20)	4 (20)	6 (30)	1 (5)	9 (45)	4 (21)	6 (31.6)	1 (5.3)	8 (42.1)
Total	8 (20)	11 (27.5)	3 (7.5)	18 (45)	9 (23.1)	11 (28.2)	2 (5.1)	17 (43.6)

As shown in **Table 2**, the concordance of attachment with respect to mother and to father was very high in these samples (96.1%, κ = 0.94). Only one child was coded as Insecure with respect to mother and Secure with respect to father (5%). On the four-way classification, all children coded as Disorganized with one parent were also Disorganized with the other parent.

Examining the differences between the two clinical groups (see **Table 2**), SSD and BDB samples did not show statistically significant differences in the attachment distribution (*p* > 0.68). Specifically, Preoccupied attachment was not more frequent in SSD subjects than in DBD subjects. In fact, the frequency of Preoccupied attachment with respect to mother was low at 5% for SSD subjects and 10% for DBD subjects. This datum is not in line with our hypothesis of a higher percentage of Preoccupied attachment for SSD subjects. Moreover, we found that the Dismissing attachment was not more frequent in the DBD sample. The frequency of the Dismissing attachment with respect to both mother and father was of 20% for BDB and 30% for SSD sample. This datum is not in line with our expectations of a wider percentage of Dismissing attachment in DBD sample.

Comparing this sample with other clinical samples (Shmueli-Goetz et al., 2008), our findings showed statistically significant differences in the attachment distribution on the four-way (*p* = 0.00) and on the presence of Disorganized attachment pattern (*p* = 0.00). Also comparisons with normative samples (Shmueli-Goetz et al., 2008) attested the presence of statistically significant differences in the attachment distribution on the three-way (*p* = 0.00).

**Table 3** reports data on attachment classifications obtained by SAT. As specified in the introduction, the critical period of middle childhood and early adolescence makes the study of attachment a continual challenge in terms of tools. The introduction of another attachment measure, the SAT (Klagsbrun and Bowlby, 1976; Attili, 2001), gives us another lens for analyzing the differences in the attachment distributions of these two clinical samples. In the overall sample of 40 clinical subjects, the two-way attachment distribution (Secure-Insecure) was the following: 52.5% was Insecure and 47.5% Secure. Disorganized attachment was found in 22.5% of the overall sample.

**Table 3:** Separation Anxiety Test (SAT’s) scores for DBD and SSD samples.

SAT subscales	DBD sample (*n* = 20)Mean (SD)	SSD sample (*n* = 20)Mean (SD)	Independent samples *t*-test
Attachment	4.35 (2.37)	3.50 (2.37)	*t*(38) = 1.13, *p* = 2.64
Loss of self-esteem	-0.30 (0.73)	-1.00 (1.65)	*t*(38) = 1.73, *p* = 0.09
Hostility	-0.30 (0.73)	-0.25 (0.55)	*t*(38) = -0.24, *p* = 0.81
Trust himself	1.00 (1.78)	1.00 (1.86)	*t*(38) = 0.00, *p* = 1.00
Avoidance	-2.20 (2.24)	-2.00 (2.97)	*t*(38) = -0.24, *p* = 0.81
Anxiety	0.75 (0.64)	0.75 (0.79)	*t*(38) = 0.00, *p* = 1.00
Anguish	-0.40 (0.82)	-0.10 (0.45)	*t*(38) = -1.43, *p* = 0.16
Confusion	-0.40 (1.05)	-0.40 (1.05)	*t*(38) = 0.00, *p* = 1.00


The presence of an over-representation of Insecure and Disorganized attachment in the overall sample confirmed our first hypothesis also with another measure of attachment. However, comparing attachment classifications on CAI and SAT, we found 22.5% of discordance on the two-way classification (Secure-Insecure). This datum suggests the importance of considering the CAI and SAT findings separately, due to the focus on different aspects of attachment.

Also with respect to this attachment measure, no significant differences were found between SSD and DBD samples. Specifically 50% of BDB and 45% of SSD were classified as Insecure. 25% of BDB and 20% of SSD were classified as Disorganized. As reported in **Table 3**, on the SAT subscales, no statistically significant differences were found between the two groups (Fisher’s Exact Test, *p* = 0.31).

### Post-Traumatic Symptomatology

In the evaluation of post-traumatic symptomatology, we hypothesized the presence of more marked levels of post-traumatic symptomatology in the SSD sample, in the areas of Anxiety, Depression, Anger, Post-Traumatic Stress, and Dissociation.

Considering the overall sample of 40 clinical subjects, children did not reach clinical cut-off on the subscales of post-traumatic symptomatology. Thus, in contrast with our hypothesis, marked levels of post-traumatic symptomatology were not reported by these samples. **Table 4** reports the differences between the two groups (*p*-values ranging from 0.01 to 0.79). No significant differences emerged on the subscales of Dissociation, Anxiety, and Post-Traumatic Stress. On the contrary, significant differences were found on Depression, Anger, and Fantasy subscales. DBD sample reported significantly higher scores on all these scales than SSD. Comparing these data with the literature (Kugler et al., 2012), our hypothesis that SSD had marked levels of Anxiety and Dissociation was not confirmed.

**Table 4:** Trauma Symptom Checklist for Children (TSCC’s) scores for DBD and SSD samples.

TSCC subscales	DBD sample (*n* = 20)Mean (SD)	SSD sample (*n* = 20)Mean (SD)	Independent samples *t*-test
Anxiety	53.00 (12.04)	50.15 (9.91)	*t*(38) = 0.82, *p* = 0.42
Depression	54.95 (11.14)	47.95 (9.05)	*t*(38) = 2.18, *p* = 0.03^∗^
Anger	60.10 (14.10)	48.85 (10.61)	*t*(38) = 2.85, *p* = 0.01^∗^
Post-traumatic stress	54.15 (13.59)	48.45 (10.52)	*t*(38) = 1.48, *p* = 0.15
Dissociation	51.80 (8.53)	48.60 (7.63)	*t*(38) = 1.25, *p* = 0.22
Overt dissociation	50.55 (8.00)	49.90 (7.71)	*t*(38) = 0.26, *p* = 0.79
Fantasy	52.90 (10.32)	46.35 (8.41)	*t*(38) = 2.20, *p* = 0.03^∗^

*^∗^p < 0.05.*

### Attachment and Post-Traumatic Symptomatology

In the evaluation of the relation between attachment and post-traumatic symptomatology, we were interested in verifying whether subjects with Disorganized attachment would present higher levels of post-traumatic symptomatology. Disorganized attachment was associated with some subscales of post-traumatic symptomatology (*p*-values ranging from 0.00 to 0.86; see **Tables 5** and **6**). Specifically, in the BDB sample, the presence of Disorganized attachment with respect to mother has been significantly linked to higher levels of Overt Dissociation (*p* = 0.00). In the SSD sample, the presence of Disorganized attachment with respect to mother has been significantly linked to higher levels of Anger (*p* = 0.02). As shown in **Table 4**, on the other subscales, the presence of Disorganized attachment would suggest higher levels of post-traumatic symptomatology, but it is not statistically significant. Findings on the relationship between attachment Disorganization with respect to father and post-traumatic symptomatology showed similar results (see **Table 6**).

**Table 5:** Relations between attachment classifications to mothers using CAI and post-traumatic symptomatology in the two clinical samples.

	Attachment classifications to mothers (four way)					
	DBD sample (*n* = 20)		SSD sample (*n* = 20)		*t*-test DBD sample	*t*-test SSD sample
TSCC subscales	Organized model	Disorganized model	Organized model	Disorganized model		
Anxiety						
Mean (SD)	54.27 (13.30)	51.44 (10.88)	46.45 (8.95)	54.67 (9.55)	*t*(18) = 0.51, *p* = 0.61	*t*(18) = -1.98, *p* = 0.06
Depression						
Mean (SD)	54.27 (13.81)	55.78 (7.40)	47.09 (11.27)	49.00 (5.77)	*t*(18) = -0.29, *p* = 0.77	*t*(18) = -0.46, *p* = 0.65
Anger						
Mean (SD)	60.64 (15.43)	59.44 (13.19)	44.18 (8.13)	54.56 (10.86)	*t*(18) = 0.18, *p* = 0.86	*t*(18) = -2.44, *p* = 0.02^∗^
PTS						
Mean (SD)	53.36 (16.22)	55.11 (10.40)	48.00 (8.90)	49.00 (12.77)	*t*(18) = -0.28, *p* = 0.78	*t*(18) = -0.20, *p* = 0.84
Dissociation						
Mean (SD)	48.64 (7.34)	55.67 (8.65)	45.64 (8.38)	52.22 (4.87)	*t*(18) = -1.97, *p* = 0.06	*t*(18) = -2.08, *p* = 0.06
Overt dissociation						
Mean (SD)	46.18 (5.19)	55.89 (7.74)	47.45 (8.08)	52.89 (6.45)	*t*(18) = -3.35, *p* = 0.00^∗∗^	*t*(18) = -1.63, *p* = 0.12
Fantasy						
Mean (SD)	53.45 (52.2)	52.22 (10.38)	43.82 (9.18)	49.44 (6.58)	*t*(18) = 0.26, *p* = 0.80	*t*(18) = -1.54, *p* = 0.12

*^∗^p < 0.05; ^∗∗^p < 0.00*.

**Table 6:** Relations between attachment classifications to fathers using CAI and post-traumatic symptomatology in the two clinical samples.

	Attachment classifications to fathers (four way)					
	DBD sample (*n* = 20)		SSD sample (*n* = 20)		*t*-test DBD sample	*t*-test SSD sample
TSCC subscales	Organized model	Disorganized model	Organized model	Disorganized Model		
Anxiety						
Mean (SD)	54.27 (13.30)	51.44 (10.88)	46.45 (8.95)	54.50 (10.20)	*t*(18) = 0.51, *p* = 0.61	*t*(17) = -1.83, *p* = 0.08
Depression						
Mean (SD)	54.27 (13.81)	55.78 (7.40)	47.09 (11.27)	48.38 (5.83)	*t*(18) = -0.29, *p* = 0.77	*t*(17) = -0.29, *p* = 0.77
Anger						
Mean (SD)	60.64 (15.43)	59.44 (13.19)	44.18 (8.13)	54.63 (13.64)	*t*(18) = 0.18, *p* = 0.86	*t*(17) = -2.31, *p* = 0.03^∗^
PTS						
Mean (SD)	53.36 (16.22)	55.11 (10.40)	48.00 (8.90)	49.13 (13.64)	*t*(18) = -0.28, *p* = 0.78	*t*(17) = -0.29, *p* = 0.83
Dissociation						
Mean (SD)	48.64 (7.34)	55.89 (7.74)	45.64 (8.38)	51.50 (4.66)	*t*(18) = -1.97, *p* = 0.06	*t*(17) = -1.78, *p* = 0.09
Overt dissociation						
Mean (SD)	46.18 (5.19)	55.89 (7.74)	47.45 (8.08)	52.75 (6.88)	*t*(18) = -3.35, *p* = 0.00^∗∗^	*t*(17) = -1.50, *p* = 0.15
Fantasy						
Mean (SD)	53.45 (10.38)	52.22 (10.84)	43.82 (9.18)	48.13 (5.62)	*t*(18) = 0.26, *p* = 0.80	*t*(17) = -1.17, *p* = 0.26

*^∗^p < 0.05; ^∗∗^p < 0.00*.

Finally, we examined the associations between SAT attachment classifications (in terms of organized attachment patterns or disorganized attachment patterns) and post-traumatic symptomatology. Findings showed significant differences only in the Dissociation subscale (*p* = 0.04) and in the Over dissociation subscale (*p* = 0.00).

Specifically, among the DBD subjects, the presence of Disorganized attachment has been significantly linked to higher levels of Overt Dissociation (*p* = 0.03). Among the SSD subjects, no significant differences have been reported (see **Table 7**).

**Table 7:** Relations between attachment classifications using SAT and post-traumatic symptomatology in the two clinical samples.

	Attachment classifications on SAT					
	DBD sample (*n* = 20)		SSD sample (*n* = 20)		*t*-test DBD sample	*t*-test SSD sample
TSCC subscales	Organized model	Disorganized model	Organized model	Disorganized model		
Anxiety						
Mean (SD)	54.27 (12.65)	49.20 (10.21)	52.00 (10.02)	42.75 (5.31)	*t*(18) = 0.81, *p* = 0.43	*t*(18) = 1.76, *p* = 0.09
Depression						
Mean (SD)	54.20 (12.11)	57.20 (8.23)	49.13 (9.72)	43.25 (2.99)	*t*(18) = -0.51, *p* = 0.61	*t*(18) = 1.17, *p* = 0.26
Anger						
Mean (SD)	59.20 (13.37)	62.80 (17.51)	50.00 (11.16)	42.25 (7.41)	*t*(18) = -0.48, *p* = 0.63	*t*(18) = 0.97, *p* = 0.35
PTS						
Mean (SD)	55.33 (13.58)	50.60 (14.52)	49.19 (11.36)	45.50 (6.40)	*t*(18) = 0.66, *p* = 0.51	*t*(18) = 0.62, *p* = 0.54
Dissociation						
Mean (SD)	49.80 (7.29)	57.80 (9.96)	47.81 (8.06)	51.75 (5.25)	*t*(18) = -1.95, *p* = 0.07	*t*(18) = -0.92, *p* = 0.37
Overt dissociation						
Mean (SD)	48.33 (6.05)	57.20 (10.08)	48.50 (7.63)	55.50 (5.80)	*t*(18) = -2.40, *p* = 0.03^∗^	*t*(18) = -1.70, *p* = 0.11
Fantasy						
Mean (SD)	52.33 (10.57)	54.60 (10.50)	46.81 (9.15)	44.50 (4.93)	*t*(18) = 0.42, *p* = 0.68	*t*(18) = 0.48, *p* = 0.64

*^∗^p < 0.05*.

## Discussion

Attachment framework has produced a solid research on the association among parenting, parent–child interaction and child development. However, a need to extend research to the middle childhood and early adolescence is a current challenge. This study aimed at adding further evidence to the complex influence of parental care on child psychopathological functioning in this age group. The aim was to evaluate children’s mental representations with respect to caregivers and post-traumatic symptomatology in two clinical samples aged 8–15 years-old: the first sample was diagnosed as having an internalizing disorder, the SSD, and the second sample was diagnosed as having an externalizing disorder, the DBD.

These samples showed a particularly interesting attachment distribution. Considering the three-way classification, which refers to the Secure, Dismissing and Preoccupied attachment, we found that Secure attachment representations with respect to mother were only 25% (28.2% with respect to father). On the contrary, Dismissing attachment representation with respect to mother was found in 50% of the samples (51.3% with respect to father). The Preoccupied classification with respect to mother was found in 25% of subjects (20.5% with respect to father). The latter attachment classification seems to be in line with the main literature assessing attachment in clinical samples while the Dismissal pattern was over-represented (Shmueli-Goetz et al., 2008; Fearon et al., 2010; Fearon and Belsky, 2011; Lecompte and Moss, 2014). Considering Disorganized attachment (with the four-way classification), we found that children with Disorganized attachment were 45% with respect to mother and 43.6% to father.

Our first hypothesis, that our risk samples were more frequently classified with Insecure and Disorganized attachment to caregivers, has been largely confirmed. This datum is in line with other studies sustaining that insecure attachment is over-represented in clinical samples, especially the Dismissing pattern (Shmueli-Goetz et al., 2008). Examining literature, in fact, it is widely attested that in normative samples the attachment distribution using CAI is around 66–64% of Secure, 30% of Dismissing, 4–6% of Preoccupied, while in clinical samples it is around 77% of insecure with respect to both mother and father, with a predominance of the Dismissing strategy (56 and 62%, respectively; Shmueli-Goetz et al., 2008). In fact, we have verified that are statistically significant differences between our sample and the Shmueli-Goetz et al. (2008) sample, where only under 10% was coded as Disorganized with at least one parent. However, comparing samples from different studies contain several limitations due to the fact that: (1) “clinical” samples are often composed by a heterogeneous group of children with different psychopathological conditions (DeKlyen and Greenberg, 2008; Shmueli-Goetz et al., 2008; Fearon et al., 2010; Fearon and Belsky, 2011); (2) in other studies focused on internalizing and externalizing disorders, attachment is not measured with CAI, but mostly with other self-report measures (e.g., Inventory of Parent and Peer Attachment; Armsden and Greenberg, 1987).

We would suggest from this datum that the evaluation of attachment models in specific psychopathological conditions is particularly useful to add information on attachment representations in middle childhood. Our distribution seems to attest the presence of a *severe clinical sample*. However, considering the attestation of the links between attachment disorganization and subsequent psychopathological outcomes, and observing findings from other studies (with lower levels of disorganization; e.g., Green et al., 2007; Shmueli-Goetz et al., 2008; Bureau et al., 2009; Borelli et al., 2010; Brumariu and Kerns, 2010; Madigan et al., 2013), further investigation on specific psychopathological conditions would be favorable.

Another surprising finding concerns the differences between the two clinical samples. Even though psychological disease is expressed in different ways, through *body* in SSD and through *acts* in DBD, these psychopathologies presented a greater similarity as compared to the state of mind with respect to attachment. Differently from the literature (Waller et al., 2004; Kozlowska and Williams, 2009), especially in SSD sample, Preoccupied attachment to caregiver was underestimated. However, it is notable that Shmueli-Goetz et al. (2008) and Zaccagnino et al. (2015) considered the low percentage of the Preoccupied classification as possibly linked to the difficulties in identifying elements of attachment preoccupation using narratives in middle childhood. Dismissing attachment had similar frequency in both samples (25% in BDB sample and 30% in SSD sample on the four-way classification). This datum is not in line with our expectations of a wider percentage of dismissing attachment among DBD subjects. In fact, the same over-representation of Dismissing attachment among SSD subjects remains to be further addressed. Nevertheless, as previously underlined, the percentage of Dismissing attachment is still high among our participants.

Due to the critical period of middle childhood and early adolescence – which the study of attachment continues to prove a challenge in terms of tools – we used a projective measure to assess attachment representation, the SAT. Findings have been in line with those of CAI, showing a wide presence of attachment insecurity in the two clinical samples. However, comparing attachment classifications on CAI and SAT, we found 22.5% of discordance on the two-way classification (Secure-Insecure). This datum showed the importance of considering the CAI and SAT findings separately, due to the focus on different aspects of attachment. In fact, it is notable that SAT is not a measure of attachment classification itself, but it captures separation anxiety from caregivers. Moreover, the classification system we used in this study (Attili, 2001) was not comparable with other SAT scoring systems where the Disorganized attachment was not identified.

Literature has indicated that attachment disorganization in clinical samples is linked to dissociative symptomatology (Cassidy and Shaver, 2008; Liotti, 2011). Thus, we were interested in exploring the association between attachment disorganization and post-traumatic symptomatology in these two clinical samples. Results showed that DBD subjects with Disorganized attachment showed significantly higher levels of Overt Dissociation than DBD subjects with other attachment patterns. SSD subjects with Disorganized attachment showed significantly higher levels of Anger. On the other aspects of post-traumatic symptomatology, the presence of Disorganized attachment classification is not particularly significant. This datum did not totally confirm our hypothesis, where we expected higher significant differences on the basis of literature (Putnam, 1997; Dutra et al., 2009; Kugler et al., 2012). For example, Kugler et al. (2012) had indicated that SSD sample would report both increased anxiety and marked levels of post-traumatic arousal symptomatology. In our samples, Post-Traumatic Stress and Dissociation did not show significant differences between DBD and SSD samples, while significant differences were found on Depression, Anxiety, and Fantasy subscales. Other studies (Dutra et al., 2009) have suggested that children who have experienced lack of parental affective involvement, may be at a particularly elevated risk for dissociation. Nevertheless, our findings did not indicate high level on dissociation in middle childhood and early adolescence. It is notable, however, that probably the aspects of relational trauma linked to dissociative symptomatology are difficult to capture combining a narratological measure of attachment representations with a self-report measure of the intensity of a perceived symptomatology. Further research to better examine this topic in middle childhood is needed. The same findings were found for the DBD sample using SAT, while in the SSD subjects no significant differences have been found.

These overall findings support that children with severe psychopathological conditions are more likely to have Insecure and Disorganized attachment in middle childhood. Literature shows how these conditions are strictly linked to unavailable care and involved in disrupted parent–child interactions (Azak et al., 2012). Thus, the quality of adult–infant interactions represents a critical context in which child adaptation problems could be evolved, and the attachment representations play an essential role in influencing child psychopathology development. It is also important to note that a mother’s insecure attachment style contributes to the understanding of variance in her estimated incompetent parenting, although it has no direct link to disorders in her offspring (Bifulco et al., 2009).

This study also suggests that, in order to better support these kinds of difficult parent–child interactions, it is necessary that intervention programs are addressed not only for the improvement of the parent–child relationship, but also the possible presence of disorganized attachment should be carefully considered. The flexibility of the children’s attachment models gives the opportunity to help parents change their own care-giving quality, encouraging reflections on children’s state of mind with respect to attachment (Regev and Snir, 2014). Nevertheless, parent management and treatments that are increasingly used in less severely damaged populations, may not be fully effective in severe clinical conditions. In the latter cases, the possible impact of disorganization may open up new important possibilities for specific modes of treatment for these families. Incidence of parental “frightening/frightened” behaviors may be reduced by powerfully reinforcing parental attention on the child in the present and encouraging reflections on children’s state of mind with respect to attachment (Solomon and George, 2011).

This work contains some evident limitations. Firstly, the sample size is a methodological limit; a bigger sample size might be useful in order to further elaborate our results. Secondly, this study does not include another measure of child symptomatology, but patients have a previous diagnosis according to DSM 5 criteria (American Psychiatric Association [APA], 2013) carried out by expert child mental health specialists. The measure of post-traumatic symptomatology with a self-report measure is probably not enough to clarify these connections. Thirdly, we have included only assessments of children in our study; considering the potential usefulness to connect child psycho-emotional condition to quality of parental care, further studies need to evaluate parents’ attachment states of mind and their psychopathological status. However, these limitations have the potential to encourage future studies on the several aspects we have highlighted.

This pilot study is the first contribution to the analysis of the role of attachment in middle childhood and early adolescence in two typical psychopathological conditions. Our findings support the role of attachment as an underlying construct for understanding child functioning given a number of psychiatric disorders. Further research on attachment disorganization could help improve clinical formulations and etiological models, and might identify a new direction in the field of family intervention programs.

## Conflict of Interest Statement

The authors declare that the research was conducted in the absence of any commercial or financial relationships that could be construed as a potential conflict of interest.
